# Epigenome-wide base-resolution profiling of DNA methylation in chorionic villi of fetuses with Down syndrome by methyl-capture sequencing

**DOI:** 10.1186/s13148-019-0756-4

**Published:** 2019-12-04

**Authors:** Ji Hyae Lim, Yu-Jung Kang, Bom Yi Lee, You Jung Han, Jin Hoon Chung, Moon Young Kim, Min Hyoung Kim, Jin Woo Kim, Youl-Hee Cho, Hyun Mee Ryu

**Affiliations:** 1 Center for Biomarker Research and Precision Medicine, CHA Advanced Research Institute, Gyeonggi-do, Republic of Korea; 2 Department of Medical Genetics, College of Medicine, Hanyang University, 222, Wangsimni-ro, Seongdong-gu, Seoul, 04763 Republic of Korea; 3 SD Genomics Co., Ltd., Seoul, Republic of Korea; 4 Department of Obstetrics and Gynecology, CHA Gangnam Medical Center, CHA University, Seoul, Republic of Korea; 5 Department of Obstetrics Gynecology, Mizmedi Hospital, Seoul, Republic of Korea; 6 Laboratory of Medical Genetics, Medical Research Institute, Cheil General Hospital and Women’s Healthcare Center, Seoul, Republic of Korea; 7 Department of Obstetrics and Gynecology, CHA Bundang Medical Center, CHA University, 59, Yatap-ro, Bundang-gu, Seongnam-si, Gyeonggi-do, 13496 Republic of Korea

**Keywords:** Down syndrome, Epigenetics, DNA methylation, Chorionic villi, Methyl-capture sequencing

## Abstract

**Background:**

Epigenetic mechanisms provide an interface between environmental factors and the genome and are influential in various diseases. These mechanisms, including DNA methylation, influence the regulation of development, differentiation, and establishment of cellular identity. Here, we performed high-throughput methylome profiling to determine whether differential patterns of DNA methylation correlate with Down syndrome (DS).

**Materials and methods:**

We extracted DNA from the chorionic villi cells of five normal and five DS fetuses at the early developmental stage (12–13 weeks of gestation). Methyl-capture sequencing (MC-Seq) was used to investigate the methylation levels of CpG sites distributed across the whole genome to identify differentially methylated CpG sites (DMCs) and regions (DMRs) in DS. New functional annotations of DMR genes using bioinformatics tools were predicted.

**Results:**

DNA hypermethylation was observed in DS fetal chorionic villi cells. Significant differences were evident for 4,439 DMCs, including hypermethylation (*n* = 4,261) and hypomethylation (*n* = 178). Among them, 140 hypermethylated DMRs and only 1 hypomethylated DMR were located on 121 genes and 1 gene, respectively. One hundred twenty-two genes, including 141 DMRs, were associated with heart morphogenesis and development of the ear, thyroid gland, and nervous systems. The genes were significantly associated with DS and various diseases, including hepatopulmonary syndrome, conductive hearing loss, holoprosencephaly, heart diseases, glaucoma, and musculoskeletal abnormalities.

**Conclusions:**

This is the first study to compare the whole-epigenome DNA methylation pattern of the chorionic villi cells from normal and DS fetuses at the early developmental-stage using MC-seq. Overall, our results indicate that the chorionic villi cells of DS fetuses are hypermethylated in all autosomes and suggested that altered DNA methylation may be a recurrent and functionally relevant downstream response to DS in human cells. This study provides basic information for future research focused on the pathophysiology of the DS and its potential effects, as well as the role DNA methylation plays in the early developmental stage of DS fetuses.

## Background

In the postgenomic era, genetic–epigenetic interactions have become a major focus. There are two main types of these interactions: *cis* interactions involve short-range effects of single-nucleotide polymorphisms and haplotypes; and *trans* interactions involve the effects of chromosomal aneuploidy on DNA methylation of CpG sites [1]. Trans-acting epigenetic effects of chromosome gain or loss lead to abnormal development and embryonic lethality. However, fetuses with Down syndrome (DS), known as the most common numerical chromosomal abnormality, survive to term. Therefore, the *trans*-acting epigenetic effects of DNA methylation are attracting increasing attention in DS research.

The estimated incidence of DS, the most prevalent genetic cause of developmental disabilities, is generally between 1 in 1000 to 1 in 1100 live births worldwide according to the World Health Organization [2]. It is characterized by over 80 clinical features that include mental retardation, stereotypical facial features, poor muscle tone, and short stature of various penetrance, and is associated with an increased risk of congenital heart disease, diabetes, leukemia, and other diseases [3]. The changes associated with DS begin early in fetal development because it is a congenital abnormality caused by the presence of an additional whole or partial copy of human chromosome 21 (HSA21). Therefore, theoretically, DS results in a 1.5-fold increased expression of genes on HSA21 and maintains genomic balance in genes on the other chromosomes. However, transcriptome analyses in DS of humans and mouse models have not revealed a strong linear correlation between the genomic imbalance of HSA21 and gene expression levels [4–8]. Transcriptomes of fetal fibroblasts from a monozygotic twin pair discordant for DS has suggested that differentially expressed genes are organized in large chromosomal domains [9]. In our prior transcriptome analysis using chorionic villi cells in the early developmental stage of DS fetuses, we also reported gene expression changes on all chromosomes, not on only HSA21 [10]. These results suggest that genes are embedded in temporally and spatially highly coordinated regulatory networks. The functional consequences of epigenetic changes, which occur at a much higher rate than DNA sequence changes (4.46 × 10–4 versus 7 × 10–9) [11], may significantly contribute to the clinical phenotype variation of DS.

In the early development stage of a DS fetus (the first or second trimester of pregnancy), a set of differentially methylated regions (DMRs) was recently demonstrated not to be enriched for HSA21 genes. Rather, these DMRs were roughly evenly distributed on all chromosomes [12–15]. Moreover, global DNA hypermethylation was demonstrated in the fetal DS cortex and placenta, with only a partial overlap of DMRs between these cell types [14, 15]. Epigenome-wide profiling in DS-based DNA methylation microarray has been performed in several tissues, including leukocytes, skin fibroblasts, buccal cells, blood, placenta, and brain [14–19]. However, these methods suffer from low genome coverage (approximately 1–3% of all CpG sites in the human genome) and introduce errors by probe cross-hybridization [20].

Here, we investigated the epigenome-wide DNA methylation patterns in the chorionic villi cells of euploid and DS fetuses using high-resolution methyl-capture sequencing (MC-seq) at a single-base resolution, which covered approximately 3.2 million CpG sites in the human genome and identified new DMRs in DS. New functional annotations of genes, including DMRs, were suggested using various bioinformatics tools.

## Results

### Overview of MC-Seq analysis of human chorionic villi cells

All chorionic villi cells were obtained in the first trimester of pregnancy (12~13 weeks of gestation, Additional file 1: Table S1). At the time of chorionic villus sampling, there were no significant differences between DS and normal groups regarding maternal age, gestational age, nuchal translucency, and gender ratio of the fetuses (*p* > 0.05 for all, Additional file 1: Table S1).

MC-seq was used to quantify DNA methylation in various CpG sites in chorionic villi cells of DS and normal fetuses. On average, 3,103,571 CpG sites with a sequencing depth > 300 in each of 10 chorionic villi samples (5 DS and 5 normal samples, matching gestational age, and fetal sex) were analyzed (Additional file 1: Table S2). Assayed CpG sites represented approximately 11% of all 28,217,449 CpG sites in the human genome (hg19), spread across regions CpG islands (CGI; 1,597,050 CpGs), CGI shores (defined as 2 kb upstream or downstream of CGIs; 810,354 CpGs), and other genomic regions (732,656 CpGs). These CpGs were distributed in the intragenic region (1,559,007 CpGs) consisting of 5′ untranslated region (UTR), exonic, intronic, and 3′UTR, and the intergenic regions (1,581,053 CpGs) including promoters (defined as a transcription start site upstream 2 kb; 820,913 CpGs), downstream (defined as a transcription termination site downstream 1.5 kb; 43,216 CpGs), and other regions (Additional file 1: Table S3).

### Higher DNA methylation levels in whole genome of the chorionic villi of fetuses with DS

**Fig. 1:**
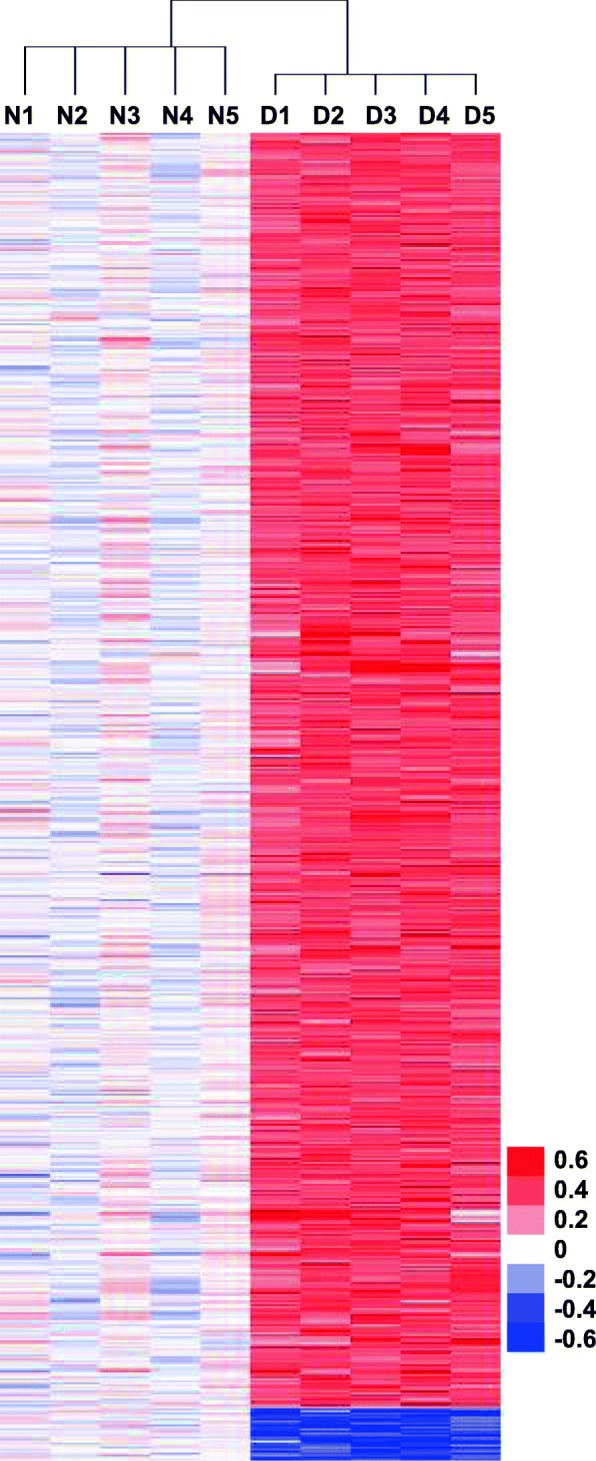
Hierarchical clustering of DMCs. The methylation degree values from MC-seq, applying independent *t* test (*p* < 0.05) and a fold-change criterion (|delta _ average of methyl degree| ≥ 0.3 in DS versus normal) produced a set of 4,439 DMCs. The *p* values were corrected using the Benjamini and Hochberg false discovery rate (FDR) method to control false positive results from multiple testing. The methylation degree values for these DMCs were subjected to hierarchical clustering. Biological samples are on the *x*-axis and DMCs are on the *y*-axis with strong methylation indicated by the red color and weak or absent methylation by the blue color. N, normal, D, Down syndrome

We analyzed DNA methylation of DS within a variety of genomic contexts of differentially methylated CpG sites (DMCs) and DMRs (see “Materials and methods” section). In DS samples, hypermethylation and hypomethylation were detected in 4,261 and 178 DMCs, respectively (Fig. 1). The number of hypermethylated DMCs far exceeded the hypomethylated DMCs in each chromosome of DS chorionic villus samples (Table 1). A comparison of the chromosome distribution of DMCs revealed that hypermethylated DMCs were most numerous on chromosome 19. Additionally, the proportional increase of hypermethylated DMCs to hypomethylated DMCs (number of hypermethylated DMC/number of hypomethylated DMC) was analyzed by chromosome size. The dominance of hypermethylated DMC was pronounced in all chromosomes and was prominent in chromosomes 8, 19, 20, and 21 (Table 1). Analysis of the distribution of DMCs according to the functional genomic regions, the dominance of hypermethylated DMCs over hypomethylated DMCs was seen in all functional genomic regions of DS (Table 2). The dominance of hypermethylated DMCs was most pronounced in the exonic part of intragenic regions and in CGI (Table 2). The average methylation levels of DMCs in promoter, CGI, and CGI shore in DS fetuses were significantly higher in those of normal fetuses (Additional file 1: Table S4).

**Fig. 2:**
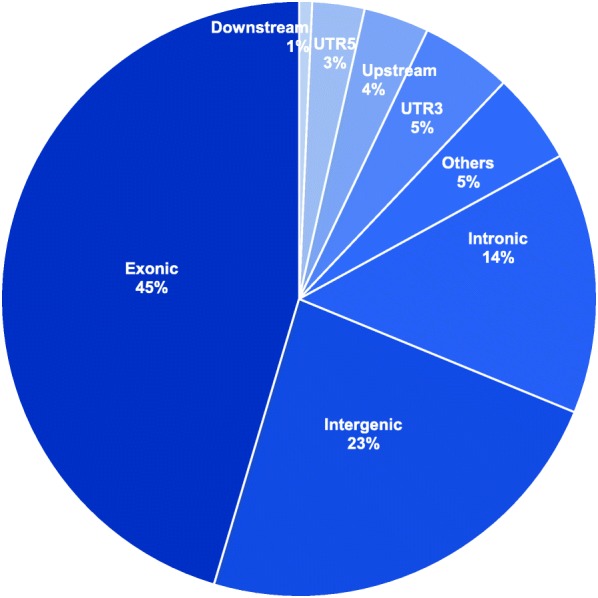
Distribution of DMRs according to the functional genomic regions

**Table 1:** Distribution of DMCs on chromosome

chr.	Size (Mb)	Hypermethlyated DMCs in DS			Hypomethylated DMCs in DS			Magnification of hypermethylated DMCs to hypomethylated DMCs	Magnification of hypermethylated DMCs to hypomethylated DMCs based on chromosome size
		n	DMCs for each chromosome of total DMCs (%)	DMCs based on chromosome size (%)	*n*	DMCs for each chromosome in total DMCs (%)	DMCs based on chromosome size (%)		
1	248.96	399	9.4	1.6	15	8.4	0.1	26.6	0.11
2	242.19	290	6.8	1.2	11	6.2	0.0	26.4	0.11
3	198.3	121	2.8	0.6	6	3.4	0.0	20.2	0.10
4	190.22	145	3.4	0.8	9	5.1	0.0	16.1	0.08
5	181.54	218	5.1	1.2	11	6.2	0.1	19.8	0.11
6	170.81	152	3.6	0.9	7	3.9	0.0	21.7	0.13
7	159.35	246	5.8	1.5	14	7.9	0.1	17.6	0.11
8	145.14	289	6.8	2.0	4	2.2	0.0	72.3	0.50*
9	138.4	93	2.2	0.7	7	3.9	0.1	13.3	0.10
10	133.8	217	5.1	1.6	7	3.9	0.1	31.0	0.23
11	135.09	259	6.1	1.9	16	9.0	0.1	16.2	0.12
12	133.28	167	3.9	1.3	9	5.1	0.1	18.6	0.14
13	114.36	99	2.3	0.9	4	2.2	0.0	24.8	0.22
14	107.04	148	3.5	1.4	3	1.7	0.0	49.3	0.46
15	101.99	156	3.7	1.5	4	2.2	0.0	39.0	0.38
16	90.34	179	4.2	2.0	13	7.3	0.1	13.8	0.15
17	83.26	182	4.3	2.2	9	5.1	0.1	20.2	0.24
18	80.37	93	2.2	1.2	4	2.2	0.0	23.3	0.29
19	58.62	476	11.2	8.1	15	8.4	0.3	31.7	0.54*
20	64.44	133	3.1	2.1	2	1.1	0.0	66.5	1.03*
21	46.71	60	1.4	1.3	2	1.1	0.0	30.0	0.64*
22	50.82	139	3.3	2.7	6	3.4	0.1	23.2	0.46
Total		4,261	100.0		178	100.0			

*Chr.* chromosome, *DMCs* differentially methylated CpG sites, *DS* Down syndrome, *Mb* megabases, *n* number

*The proportional increase of hypermethylated DMCs to hypomethylated DMCs is 0.5 or more, depending on the chromosome size. It means that these chromosomes were more enriched with hypermethylated CpG sites.

**Table 2:** Distribution of DMCs on functional genomic regions

Functional genomic regions	Hypermethlyated DMCs		Hypomethylated DMCs		Magnification of hypermethylated DMCs to hypomethylated DMCs
	*n*	%	*n*	%	
Intragenic	2,539	57.2	100	2.3	25.4
5′UTR	98	2.2	2	0.0	49.0
3′UTR	146	3.3	10	0.2	14.6
Intronic	905	20.4	66	1.5	13.7
Exonic	1,390	31.3	22	0.5	63.2
Intergenic	1,216	27.4	49	1.1	24.8
Promoter	488	11.0	22	0.5	22.2
Downstream	60	1.4	0	-	-
CGI	3,053	68.8	59	1.3	51.7
CGI shore	499	11.2	23	0.5	21.7

*CGI* CpG island, *DMCs* differentially methylated CpG sites, *UTR* untranslated region

Percentages of DMCs on functional genomic regions was measured based on total 4439 DMCs

### The common change of DNA methylation observed in fetal DS

To further investigate the common changes of DNA methylation observed in fetal DS regardless of tissue type, we compared the DNA methylation data from DS fetal brain tissue with these results. A publicly available dataset of fetal brains (GEO #: GSE73747) was reanalyzed according to the criteria of DMC selection used in this study. In fetal brains with DS, eight DMCs were hypermethylated and only one DMC was hypomethylated, compared to the methylation patterns in normal fetal brains (Additional file 1: Table S5). Of the DMCs of the brains and the chorionic villi of fetal DS, *CPT1B* on chromosome 22 and *TNXB* on chromosome 6 were commonly hypermethylated, regardless of tissue type, but involved none of hypomethylated DMCs. To confirm the DNA methylation pattern of *CPT1B*, we performed methylation-specific quantitative real-time polymerase chain reaction (PCR). The ΔCt value of *CPT1B* was significantly lower in the DS than normal (Table 3). It means that the gene was significantly hypermethylated in the chorionic villi cells of DS fetuses, compared with those in normal fetuses. The DNA methylation pattern of the gene was consistent with that of MC-seq.

**Table 3:** Ct values of genes using methylation specific quantitative real-time PCR

Target gene	*N* (*n* = 10)			DS (*n* = 8)			*p* value
	MSRE (−)	MSRE (+)	ΔCt	MSRE ((−)	MSRE (+)	ΔCt	
*CPT1B*	32.73	35.31	2.58	33.35	33.55	0.19	0.033
*ZNF837*	35.47	37.59	2.13	36.77	36.61	0.21	0.033
*C11orf95*	29.62	33.74	4.12	30.02	30.74	0.72	0.006
*KIAA1875*	30.55	41.94	11.59	34.37	35.65	2.89	0.007
*SHROOM1*	30.07	34.12	4.05	29.86	30.73	0.86	0.001
*CASZ1*	34.00	37.83	3.27	34.33	35.19	0.85	0.008

*Ct* cycle threshold, *DS* Down syndrome, *N* normal, *MSRE* methylation-specific restriction enzyme

### Validation of MC-Seq analysis by methylation-specific quantitative real-time PCR

We selected 141 DMRs from the total of 4439 DMCs based on criteria for DMR selection (Additional file 1: Table S6). All DMRs, excluding one, were hypermethylated in the whole genome. We selected the top five DMRs (*ZNF837*, *C11orf95*, *KIAA1875*, *SHROOM1*, and *CASZ1*) and confirmed their DNA methylation patterns using methylation-specific quantitative real-time PCR. The DNA methylation patterns of the DMRs were consistent with that of MC-seq (Table 3). The largest number of DMRs was present on chromosome 19, as observed for DMCs (Additional file 1: Table S7). Most hypermethylated DMRs were presented in the exonic region of intragenic regions (Fig. 2). One hundred twenty-two genes including DMRs were used for the functional annotation analysis related to DS.

### Significant influence of DMRs in biological process and disease associations of DS

In the in silico analysis using the 122 DMR genes, gene ontology (GO) annotation and disease association analyses were performed using a statistical hypergeometric test, with a significance level of *p* < 0.05 and a minimum of three genes. In the biological process category of GO annotation (Table 4), regulation of heart morphogenesis was the most significant (adj*P =* 0.0050). Related DMR genes were *SLX1*, *WNT3A*, *TBX1*, and *SOX9*. The largest number of DMR genes (*n* = 34) involved in system development (adj*P =* 0.0084). Significant correlations were evident for the development of the ear, thyroid gland, and nervous system (adj*P* < 0.05 for all). The glutamate receptor signaling pathway and regulation of ion transport were also significantly related to the DMR genes (adj*P* < 0.05 for both). In the molecular function category of GO annotation (Table 4), *KCNA5*, *SHANK1*, and *CACNA1H* were most significantly associated with the scaffold protein binding (adj*P =* 0.0143). The largest number of genes (*n* = 13) was involved in sequence-specific DNA binding transcription factor activity (adj*P =* 0.0388). However, there was no significant association with the cellular component category of GO annotation. The disease associations of the DMR genes are shown in Table 5. The most statistically significant associations were for hepatopulmonary syndrome and conductive hearing loss (for both; adj*P =* 0.0039). The largest number of genes (*n* = 7) was involved in congenital abnormalities (adj*P =* 0.0224). DMR genes were also significantly associated with DS, craniofacial abnormality, heart disease, chromosome aberrations, musculoskeletal abnormalities, and genetic translocation (adj*P <* 0.05 for all).

**Table 4:** GO analysis of identified DMR genes

	Pathway	GeneSymbol	rawP	adjP
BP	Regulation of heart morphogenesis	*SIX1 WNT3A, TBX1, SOX9*	5.63e-06	0.0050
	Glutamate receptor signaling pathway	*MINK1, GRIN3B, SHANK1, MAPK8IP2*	0.0001	0.0074
	Organ morphogenesis	*SIX1, LEFTY1, SIX6, TBX1, SOX9, AXIN2, DUOX2, UNCX, WNT3A, TLX1, F7, MYO15A, FOXL1, KIF26B*	0.0001	0.0074
	Nervous system development	*MINK1, SLC8A3, SOX9, CACNA1H, STMN3, UNCX, TLX1, SEPP1, FOXL1, MAPK8IP2, FOXD2, RTN1, SIX1, TACC2, TBX1, SHANK1, MNX1, DUOX2, SALL3, WNT3A, BRSK2, CRMP1, PURA*	3.20e-05	0.0074
	Anatomical structure morphogenesis	*MINK1, LEFTY1, SIX6, SOX9, SHROOM1, CACNA1H, UNCX, TLX1, MYO15A, FOXL1 MAPK8IP2, FOXD2, KIF26B, RSG1, SIX1, TBX1, SHANK1, MNX1, AXIN2, DUOX2, SALL3, ECE1, WNT3A, BRSK2, F7, CRMP1*	2.06e-05	0.0074
	Central nervous system development	*SIX1, SLC8A3, TACC2, TBX1, MNX1, SOX9, DUOX2, UNCX, SALL3, WNT3A, TLX1, SEPP1, FOXL1*	8.41e-05	0.0074
	Ear development	*DUOX2, SIX1, ECE1, WNT3A, MYO15A, TBX1, SOX9*	6.29e-05	0.0074
	System development	*MINK1, LEFTY1, SIX6, SOX9, CACNA1H, TLX1, AHSP, SEPP1, RTN1, SIX1, ZNF354A, CBFA2T3, TACC2, SHANK1, MNX1, AXIN2, SALL3, ECE1, WNT3A, SLC8A3, STMN3, UNCX, MYO15A, FOXL1, MAPK8IP2, KIF26B, FOXD2, TBX1, DUOX2, HLA-DOA, BRSK2, CRMP1, F7, PURA*	0.0002	0.0084
	Thyroid gland development	*DUOX2, SIX1, TBX1*	0.0002	0.0084
	Otic vesicle development	*SIX1, TBX1, SOX9*	0.0002	0.0084
	Inner ear morphogenesis	*SIX1, WNT3A, MYO15A, TBX1, SOX9*	0.0002	0.0084
	Inner ear development	*DUOX2, SIX1, WNT3A, MYO15A, TBX1, SOX9*	0.0002	0.0084
	Regulation of ion transport	*KCNA5, MINK1, DPP6, GRIN3B, SHANK1, MAPK8IP2, BEST1*	0.0006	0.0133
MF	Scaffold protein binding	*KCNA5, SHANK1, CACNA1H*	0.0001	0.0143
	RNA polymerase II distal enhancer sequence-specific DNA binding transcription factor activity	*PURA, FOXL1, SOX9, FOXD2, NFIX*	0.0002	0.0143
	Sequence-specific DNA binding transcription factor activity	*SIX1, ZNF354A, CBFA2T3, SIX6, TBX1, MNX1, SOX9, UNCX, TLX1, PURA, FOXL1, FOXD2, NFIX*	0.0019	0.00388

*BP* biological process, *MF* molecular function, *rawP p* value from hypergeometric test, *adjP p* value adjusted by the multiple test adjustment

**Table 5:** Disease association of identified DMR genes

Disease	GeneSymbol	rawP	adjP
Hepatopulmonary syndrome	*KCNA5, DUOX2, ECE1, THBD*	8.40e-05	0.0039
Hearing loss, conductive	*TECTA, SIX1, MYO15A, TBX1*	8.01e-05	0.0039
Holoprosencephaly	*SIX6, GDF1, MNX1*	0.0002	0.0062
Craniofacial abnormalities	*WNT3A, TBX1, GDF1, SOX9, AXIN2*	0.0011	0.0187
Down syndrome	*RAB9BP1, SUMO3, ANKRD20A11P, HLA-DOA, EFEMP2*	0.0011	0.0187
Heart diseases	*KCNA5, F7, TBX1, GDF1, THBD*	0.0033	0.0224
Chromosome aberrations	*TLX1, CBFA2T3, TBX1, MNX1, SOX9*	0.0035	0.0224
Testicular diseases	*ALPPL2, YJEFN3, SOX9*	0.0027	0.0224
Congenital abnormalities	*SIX1, SIX6, TBX1, GDF1, MNX1, SOX9, AXIN2*	0.0019	0.0224
Hearing loss, non-syndromic	*TECTA, SIX1, MYO15A*	0.0029	0.0224
Glaucoma	*SIX1, SIX6, BEST1*	0.0021	0.0224
Musculoskeletal abnormalities	*TBX1, GDF1, MNX1, SOX9, AXIN2*	0.0020	0.0224
Translocation, genetic	*TLX1, SLC8A3, CBFA2T3, MNX1, SOX9*	0.0065	0.0290

*BP* biological process, *MF* molecular function, *rawP p* value from hypergeometric test, *adjP p* value adjusted by the multiple test adjustment

## Discussion

The mechanisms underlying congenital impairment in DS are unclear. Accumulating evidence suggests that DS phenotypes, including various pathological changes, occur by alterations of the complex regulation of many genes on and outside HSA21. The alterations could affect the roles of the upstream effector genes, located on HSA21, and the downstream target genes that are affected by DNA methylation, which are distributed throughout the genome of DS individuals. Therefore, investigating the epigenomic changes that contribute to the various phenotypes of DS may improve our understanding of the pathophysiology of DS.

In the current study, we performed DNA methylation profiling at single-base resolution in the chorionic villi cells of human DS and euploid fetuses at an early developmental stage. Global DNA hypermethylation across the whole epigenome was evident in DS fetuses, with methylation differing in 4439 DMCs compared to that in euploid fetuses. Most DMCs were hypermethylated in DS and only approximately 4% of DMCs were hypomethylated. These DMCs were distributed in all chromosomes, regardless of the methylation pattern in DS. Hypermethlyated DMCs were pronounced in the exonic regions in intragenic regions and the promoters in intergenic regions. Additionally, two genes (*CPT1B* and *TNXB*) were commonly hypermethylated in fetal brain and chorionic villi in DS. We identified 141 DMRs that ranged from three to 79 DMCs. All DMRs, except one DMR, were hypermethylated in DS. In a functional annotation of identified DMR genes, we found that these genes with hypermethylated DMRs were significantly related to the various phenotypes and pathophysiology including chromosomal aberration and congenital abnormalities of DS. This finding suggests the possibility of fetal epigenetic therapy by inhibiting the global DNA methylation in DS. Furthermore, chorionic villi cells (e.g., trophoblasts) are the major origin of cell-free fetal DNA in maternal blood [21]. Therefore, profiling DNA methylation in chorionic villi cells of fetal DS could lead to potential clinical application to develop novel approaches for the noninvasive prenatal test of DS in the first trimester of pregnancy by analysis of fetal DS-specific DNA methylation in maternal blood.

Global hypermethylation in DS was conserved in different tissue types and developmental stages [13–19]. However, the number and location of DMCs differed in the whole genome depending on tissue types and developmental stage. In peripheral blood leukocytes of DS adults*,* eight genes (*TMEM131*, *TCF7*, *CD3Z/CD247*, *SH3BP2*, *EIF4E*, *PLD6*, *SUMO3*, and *CPT1B*) were hypermethylated [18]. In the fetal cortex of DS, nine genes (*C1orf35*, *CPT1B*, *DECR2*, *FAM83H*, *GLI4*, *LRRC14*, *LRRC24*, *STK19*, and *TNXB*) were hypermethylated [14]. In the fetal placenta of DS, hypermethylation of *CPT1B*, *TCF7*, and *FAM62C* was evident [15]. We also found hypermethylation in the 5′UTR of *CPT1B* in chorionic villi cells at an early developmental stage of fetal DS. These results suggest that hypermethylation of *CPT1B* is conserved in various tissue samples of the different developmental origin of DS and may be a common epigenetic characteristic in DS. *CPT1B* on chromosome 22 is one of the carnitine palmitoyltransferase 1 genes [22]. This enzyme is required for the net transport of long-chain fatty acyl-CoAs from the cytoplasm to the mitochondria [14, 22]. Mitochondrial dysfunction has been found in the brains of patients with various psychiatric disorders, including DS [14]. Placental mitochondrial dysfunction may also be critical in a range of gestational disorders, which has important implications for maternal and fetal/offspring health. Therefore, these results provide the new insight that the hypermethylation of *CPT1B* may be associated with mitochondrial dysfunction in the developing DS.

In this study, the methylation degree of *ZNF837*, *C11orf95*, *KIAA1875*, *SHROOM1*, and *CASZ1* with more than 40 hypermethylated DMCs were confirmed by methylation-specific real-time PCR. The results were consistent with the MC-seq results. Among these hypermethylated DMR genes, *SHROOM1* and *CASZ1* appear important potentials in pathophysiology of complications related to DS. *SHROOM1* on chromosome 5 encode Shroom Family Member 1. SHROOM family members play diverse roles in the development of the nervous system and other tissues and may be involved in the assembly of microtubule arrays during cell elongation [23]. Diseases associated with SHROOM1 include atrial septal defect 2 and atrial heart septal defect. GO annotations related to this gene include actin filament binding. Ectopic expression of SHROOM1 elicited apical accumulation of gamma-tubulin in naive epithelial cells, consistent with a role for SHROOM1 in governing microtubule architecture [24]. It was recently reported that patients with knee osteoarthritis had marked hypermethylation status in all *SHROOM1* differentially methylated sites between damaged and non-damaged tissues [25]. The authors suggested hypermethylated *SHROOM1* as a promising candidate for functional studies of osteoarthritis pathology [25]. In another study, hypermethylation of *SHROOM1* was present in DS fetuses with or without congenital heart defects, as well as in fetuses with heart malformations [16]. Congenital heart defects are the most common malformation occurring in DS. There is also an increased risk of skeletal abnormalities, including osteoarthritis, in DS. There certainly is a higher incidence of heart and joint problems in DS. However, these disorders are thought to have a multifactorial etiology and the main causes are largely unknown. Therefore, hypermethylation of *SHROOM1* in DS may be potentially associated with the pathophysiology of the congenital heart defects and osteoarthritis related to DS. Additionally, *CASZ1* on chromosome 1 is a protein-coding gene of castor zinc finger 1, which is a zinc finger transcription factor. The single-nucleotide polymorphisms in this gene are associated with blood pressure variation. Alternative splicing yields multiple transcript variants encoding different protein isoforms [26]. They are involved in vascular assembly and morphogenesis through direct transcriptional regulation of *EGFL7* [27]. Diseases associated with *CASZ1* include retroperitoneal sarcoma and retroperitoneum carcinoma. Interestingly, *Casz1* is expressed in a number of developing tissues, including neural, endothelial, and cardiac tissues. Improper regulation of CASZ1 could potentially lead to the defects that are collectively observed in DS, such as cognitive defects, congenital heart defects, and hypertension [28, 29]. CASZ1 also has an established role in regulating cellular adhesion in multiple tissues. In previous studies of CASZ1 function, HSA21 resident-congenital heart disease 5 protein (CHD5) was demonstrated as being interactive and essential protein for CASZ1 function, with the CHD5-CASZ1 interaction being necessary for cardiac morphogenesis [30, 31]. Therefore, a potential mechanism that could link the misregulation of CASZ1 to one or more pathologies related to DS has been suggested. However, the precise mechanism by which CASZ1 is regulated by its interaction with CHD5 has yet to be determined. In this study, we firstly found that exonic region of *Casz1* is hypermethylated in DS. This epigenetic change of *Casz1* in DS may provide additional insight into the phenotypes generated by its misregulation. Furthermore, *ZNF837* on chromosome 19 encodes zinc finger protein 837 and is commonly hypermethylated in the adult and fetal brain, and the fetal placenta in DS [1]. In this study, *ZNF837* was identified as the DMR gene with the largest DMCs in the chorionic villi cells in the early developmental stage of DS fetuses. Although this gene may be involved in transcriptional regulation, since ZNF837 is located in the nucleus, an obvious function is still unclear. *C11orf95* (*Chromosome 11 Open Reading Frame 95*) is associated with ependymoma as tumors of the brain and spinal cord [32]. C11orf95–RELA fusion protein translocates spontaneously to the nucleus to activate nuclear factor-kappa B target genes, and rapidly transform neural stem cells—the originating cells of ependymoma—to form these tumors [33]. However, ependymoma in DS has only been reported so far in only two cases, and the association of ependymoma with DS is a rarity [34, 35]. Finally, *KIAA1875* (previous HGNC symbols for *WDR97* and encode WD Repeat Domain 97) has an unknown function and protein-coding potential [36]. Further research is needed to understand the epigenetic function and mechanisms of known and still-undiscovered DMR genes. Our results have potential utility value as basic information for further studies of these DMR genes in DS.

Several studies have compared the DNA methylation profiles between normal and DS patients and have demonstrated common epigenetic characteristics. These include global hypermethylation and a lack of enrichment on HSA21. This DNA methylation pattern may be tissue and developmental stage-specific in DS. Moreover, DS as a congenital aneuploidy may involve continuous epigenetic changes in whole genome from embryos early in development. These early epigenetic changes may be the basis of the developmental defects in DS. Therefore, it is important to investigate epigenetic changes in the early development of DS fetuses to clarify the pathogenesis of DS. Recently, global hypermethylation was demonstrated in the fetal cortex and placenta of human DS [14, 15]. We also found the same methylation pattern in early chorionic villi cells of fetal sex and gestational age-matched DS and normal fetuses. However, the percentage of hypermethylated DMCs identified in each study varied from 1.85 to 4%. The variation likely reflects the impact of various factors, such as tissue type, gestational age at sampling, criteria for DMC selection, and methods used. In this study, we used MC-Seq for high-throughput methylome profiling in DS. This is a next-generation sequencing approach that targets specific loci in the epigenome. MC-seq utilizes target- specific bait sequences, circumventing this restriction through bait design and allows for epigenome-wide surveying of specific genomic loci of physiological and clinical interest. Thus, it presents an attractive, cost-effective alternative to uncover novel disease-associated genomic loci. Moreover, MC-seq overcomes the limitations of lower genome coverage in a methylation microarray, high cost, and processing time in whole-genome bisulfite sequencing, while avoiding overrepresentation in next-generation sequencing platforms, such as reduced representation bisulfite sequencing and methylated DNA immunoprecipitation sequencing [20]. Furthermore, in this study, the MC-seq with a depth exceeding 300× was performed to overcome the technical variation in detecting small inter-individual/inter-group differences. Therefore, we were available to interrogate DNA methylation more precisely in clinical samples at the single-base resolution. However, many of our results were based on databases of bioinformatics tools. Although these in silico bioinformatic tools are useful to predict multi-genes associated with the pathophysiology of disorder, the in silico results are not strong enough to justify the functional significance of genes. Moreover, due to the very small amount of fetal chorionic cells that were obtained, we could not gain more confidence through further studies of gene expression in DS. Therefore, the functional roles of the potential pathways in our study need further characterization, possibly in cell lines or tissues relevant to specific DS phenotypes. These potential pathways with epigenetic changes in DS remain to be further elucidated. Furthermore, this study was limited by its small sample size and no adjustment for confounders. DNA methylation could be affected by various environmental and clinical factors. Therefore, future, larger studies are needed to substantiate our findings. Despite the limitations of this study, our findings warrant further studies addressing DNA methylation of the whole genome associated with the pathogenesis of DS. Various further studies might support the functional significance of our new insight into the pathophysiology of DS.

## Conclusion

To our knowledge, this is the first study to perform high-throughput methylome profiling of the chorionic villi cells of DS fetuses using MC-seq. Various CpG sites across the whole genome were differentially methylated between euploid and DS fetuses, and most were hypermethylated in DS. Furthermore, our results indicate that many biological pathways implicated in DS and its complications may be regulated by genes with DMRs. Therefore, the current study provides a variety of information that may give a better understanding of epigenetic modulations in whole genome of DS.

## Material and methods

### Study subjects

The Institutional Review Board of Cheil General Hospital approved this study (#CGH-IRB-2016-5). Singleton pregnant women who attended antenatal care at the hospital’s Department of Obstetrics and Gynecology between June 2015 and May 2017 were enrolled for this study. Written informed consent was obtained from all patients under the approval from the IRB. Chorionic villi cells from subjects were obtained in the first trimester of pregnancy (12 ~ 13 weeks of gestation, Additional file 1: Table S1) and stored in liquid nitrogen until analysis. The gestational age of each fetus was determined by ultrasonography.

Chromosomal analyses using the GTG banding method were performed to determine the karyotypes of fetal chorionic villi cells. All DS samples were a complete extra copy of HSA21 (47, XX, +21 or 47, XY, +21) and all controls showed normal karyotypes (46, XX or 46, XY) (Additional file 1: Table S1). The causes for chromosomal analyses were maternal advanced age and increased nuchal translucency (Additional file 1: Table S1). The cases and controls were matched for the gender ratio of fetuses. The genomic DNA of each sample was extracted from chorionic villi cells using the QIAamp DNA Mini Kit (Qiagen, catalog no. 51304), according to the manufacturer’s instructions.

### High-throughput methylome profiling using MC-seq

Standard DNA methylation region capture libraries were generated using the SureSelect Methyl-Seq Target Enrichment protocol (Agilent) for a paired-end sequencing library (ver. B.3, June 2015; Illumina) with 3 μg genomic DNA. In all cases, the SureSelect Human Methyl-Seq probe set (Agilent, catalog no. 5190-4662) was used. The quantification of DNA and the DNA quality was done using measured by PicoGreen assay kit (Thermo Fisher Scientific, catalog no. P7589) and Nanodrop spectrophotometer (NanoDrop Technologies, catalog no. ND-2000), respectively.

Fragmentation of the 3 μg of genomic DNA was performed using adaptive focused acoustic technology (AFA; Covaris, catalog no. 500219) to a target peak size of 150–200 bp. Briefly, the eight microTUBE Strips were loaded into the tube holder of the ultrasonicator and shear the DNA using the following settings: mode, frequency sweeping; duty cycle, 10%; intensity, 5; cycles per burst, 200; duration, 60 s × 6 cycles; temperature, 4 °C ~ 7 °C. The fragmented DNA is repaired, an “A” is ligated to the 3′ end, SureSelect Methyl-Seq Methylated Adapter are then ligated to the fragment. Once ligation was assessed, the adapter ligated product was PCR-amplified. The final purified product such as the methylated adapter-ligated DNA was then quantified according to the qPCR Quantification Protocol Guide and qualified using the TapeStation DNA screen tape D1000 (Agilent, catalog no. 5067-5582).

For DNA methylation region capture, 350 ng of DNA library was mixed with hybridization buffers, blocking mixes, RNase block, and 5 μl of the SureSelect All DNA methylation region Capture Library, according to the standard SureSelect Methyl-Seq Target Enrichment protocol (Agilent). Hybridization to the capture bait was conducted at 65 °C using a heated thermal cycler lid option at 105 °C for 24 h with a PCR machine. Target captured DNAs were treated with bisulfite using the EZ DNA Methylation-Gold Kit (Zymo Research, catalog no. D5005), subjected to eight PCR cycles to enrich adaptor added fragments, and six PCR cycles to add multiplexing barcodes. The captured DNA was then amplified. The final purified product was quantified using the aforementioned qPCR Quantification Protocol Guide and qualified using the TapeStation DNA screen tape D1000 (Agilent, catalog no. 5067-5582). Sequencing was done using the HiSeq™ 2500 platform (Illumina, catalog no. SY–401–2501).

### Data processing and methylation profile calling

The quality of the paired-end raw reads generated from sequencing was checked using FastQC software (version 0.11.5). Before starting the analysis, Trimmomatic (version 0.32) was used to remove adapter sequences and bases with a base quality < 3 from the end reads. Also, using the sliding window trim method, bases that did not qualify for window size = 4, and mean quality = 15 were removed. Reads with a minimum length of 36 bp were removed to produce cleaned data. The cleaned reads were aligned to the *Homo sapiens* genome (UCSC hg19) using the bisulfite sequencing MAPping program (BSMAP; version 2.90 parameter set −n 1 −r 0) based on the Short Oligo Alignment program (SOAP), which allowed only uniquely mapped reads. Mapped data in SAM file format were sorted and indexed using SAMtools (version 1.2). PCR duplicates were removed with sambamba (version 0.5.9).

The methylation level was determined with the methratio.py feature in the BSMAP program [37]. The methylation ratio of every single cytosine located in the Agilent SureSelect target region satisfying higher than 10 CT count was reliable generally as a methylation call. For the regions covered by both ends of a read pair, only one read was used to call methylation. The resulting coverage profiles were summarized as # of C/effective CT counts for each of the three-sequence context (CG, CHG, and CHH).

### Identification of DMCs and DMRs

The methylation level at each base of CpG was normalized with median scaling normalization. An independent *t* test was used to assess the significance of methylation differences between the two groups for five comparison pair. The *p* values were corrected using the Benjamini and Hochberg false discovery rate (FDR) method to control false-positive results from multiple testing as our prior study [10]. The principal component analysis revealed the separation of samples based on disease status (normal or DS) but not fetal gender, as a prior study [15].

DMCs were determined by filtering each region associated with |delta_mean| ≥ 0.3, independent *t* test *p* values < 0.05, and FDR < 0.05. DMRs were defined as contiguous regions of any length containing ≥ 3 DMCs. Hierarchical clustering analysis also was performed using complete linkage and Euclidean distance as a measure of similarity to display the methylation level of samples for significant CpGs, which satisfied at least one more comparison pair. The heatmap was plotted automatically by a centroid linkage using the centered absolute correlation in the similarity metric. All data analysis and visualization of the differentially methylated result was conducted using R 3.3.1 (www.r-project.org) and Statistical Package for Social Sciences 12.0 (SPSS Inc.).

### Comparison of DS fetal brain

Microarray data for the brain of DS and control subjects were downloaded from the Gene Expression Omnibus (GEO, http://www.ncbi.nlm.nih.gov/geo/). A publicly available microarray dataset of human DS fetal brains (GEO #GSE73747) was used. The dataset was reanalyzed according to the aforementioned DMC and DMR criteria. We used data of 61 fetal brain samples (36 normal fetal brains, 25 DS fetal brains).

### Methylation-specific quantitative real-time PCR of DMRs

We confirmed the methylation level of the MC-seq data using methylation-specific quantitative real-time PCR. The genomic DNA of each sample was isolated from the chorionic villi cells of normal (*n* = 10) and DS (*n* = 8) fetuses using the QIAamp DNA Mini Kit (Qiagen, catalog no. 51304). Samples that had been used for the MC-seq were also included (normal DS = 5:4). DNA samples (10 ng) were treated with AciI (New England Biolabs, catalog no. R0551L) as the methylation-specific restriction enzyme (MSRE) according to the manufacturer’s instructions, and then concentrated to 40 μL using the DNA Clean and Concentrator (Zymo Research, catalog no. D4031). Through this process, unmethylated DNA including the recognition sequence of MSRE was cleaved. The MSRE-treated DNA and untreated DNA were amplified by real-time PCR. The sequences of PCR primers used and PCR conditions are presented in Additional file 1: Table S8.

For the analysis of methylation levels of DMRs, the delta (Δ) threshold cycle (Ct) value was calculated as ΔCt = Ct_MSRE−_Ct_input_. Input indicates the level of the target gene in whole genome without MSRE treatment and MSRE indicates the level of the methylated target gene that remained after MSRE treatment. Therefore, the ΔCt value represented the methylation level of the target gene. The smaller the ΔCt value, the higher the methylation level of the target gene. The *p* value of the study subjects was analyzed using the Mann-Whitney *U* test and a *p* value < 0.05 was considered statistically significant. Statistical analyses were performed using the Statistical Package for Social Sciences 12.0 (SPSS Inc.).

### Functional annotation of DMR genes

The lists of genes with DMRs were submitted to a functional annotation tool provided by WebGestalt (http://www.webgestalt.org/webgestalt_2013/). Gene ontology (GO) analysis and disease-associated gene analysis were performed according to criteria of a statistic hypergeometric test with a significance level of adj*P* < 0.05, a Benjamini and Hochberg multiple test adjustment, and a minimum of three genes [10].

## Supplementary information

**Additional file 1: Table S1.** Clinical characteristics. **Table S2.** MC-seq performance of each sample. **Table S3.** CpG sites of each sample covered by MC-seq. **Table S4.** Methylation levels of DMCs in the functional genomic regions. **Table S5.** Methylation levels of DMCs in the fetal normal and DS brain. **Table S6.** Genes with DMRs. **Table S7.** Functional region and chromosomal location of genes with DMRs. **Table S8.** Primer sequences and PCR condition for methylation specific quantitative real-time PCR .

## Data Availability

Not applicable.

## Abbreviations

CGI: CpG islands
Ct: Threshold cycle
DMCs: Differentially methylated CpG sites
DMRs: Differentially methylated regions
DS: Down syndrome
GO: Gene ontology
HSA21: Human chromosome 21
MC-Seq: Methyl-capture sequencing
MSRE: Methylation-specific restriction enzyme
